# Supplementary material for: Local adaptation and coping strategies to global environmental changes: Portraying agroecology beyond production functions in southwestern Ethiopia
Source: PLoS One. 2021 Aug 12;16(8):e0255813. doi: 10.1371/journal.pone.0255813 (PMC8360511; doi:10.1371/journal.pone.0255813)
Supplement: S1 Text — (DOCX) [file pone.0255813.s001.docx]

**S1 Text. English language survey questionnaire developed to collect data from small-scale farmers household heads to appraise local adaptation and coping strategies to global environmental changes across agroecology in Southwestern Ethiopia (DOCX)**

Dear respondent your household has been randomly selected to participate in this research. Your genuine responses are extremely important for success of this research. I assure you that your answers will be completely confidential, summary information will be used for analysis, and no individual questionnaire will be made available to any authority.

| Date of interview | ____________________ | Questionnaire No. | ________________ |
| --- | --- | --- | --- |
| District | ­­­____________________ | Kebele | ________________ |

**Part I: Households Socio-economic and Demographic Characteristics**

|  | Gender of the household head | | | | | (1) Male | | | | | | | | | | (2) Female | | | | |
| --- | --- | --- | --- | --- | --- | --- | --- | --- | --- | --- | --- | --- | --- | --- | --- | --- | --- | --- | --- | --- |
|  | Age of the household head | | | | | (1) 18 – 30yrs  (2) 31 – 45yrs | | | | | | | | | | (3) 46-65  (4) 65+ | | | | |
|  | How long have you lived in this kebele? | | | | | | | (1) <5 years  (2) 5-10 years | | | | | | | | (3) 11-15 years  (4) Over 16 years | | | | |
|  | What is your household size? | | | (1) < 4 persons  (2) 4– 7 persons | | | | | | | | | | (3) 8– 15 persons  (4) >16 persons | | | | | | |
|  | Highest level of education for the household head | | | (1) No formal education  (2) Less than Grade 5 | | | | | | | | | | (3) Grade 5-8  (4) Secondary & above (> 9)  (5) Tertiary level | | | | | | |
|  | What is your marital status? | | | | | (1) Single  (2) Married | | | | | | | | | | (3) Divorced  (4) Widowed | | | | |
|  | Of the household, the extent of dependence (number of persons below 15 years out of total house hold size) | | | | | | | | | | | | (1) ≤ quarterly  (2) nearly half | | | | | (3) more than half | | |
|  | Do you have relatives in this area, how many? | | | | | | | | | | (1) No  (2) < 5 households | | | | | | | | (3)>5 households | |
|  | Have you any socio-economic/ customary institutions? | | | | | | | | | | (1) No | | | | (2) Yes | | | | | |
|  | If yes on Q. 9, How many institutions? ---------- | | | | | | | | | | | | | | | | | | | |
|  | Do you have access to extension services? | | | | | | | | | (1) No | | | | | (2) Yes | | | | | |
|  | If ‘yes’ for Q.11, how did they visit you in a year? | | | | 1. Once in a year 2. Every quarterly | | | | | | | | | | 1. Twice in a year 2. Every month in a year | | | | | |
|  | How many years of experiences do you have on your main livelihoods? | | | | | | | | | | (1) < 3 years  (2) 3-10 years | | | | | (3) > 10 years | | | | |
|  | Do you have access to land management and climate change information? | | | | | | | | | | | | | | | (1) No (2) Yes | | | | |
|  | What is your main source of income? | | | | | | 1. farm based 2. off-farm | | | | | | | | | (3) Non-farm  (4) All | | | | |
|  | If you selected 1 on Q. 15, what type of farming system do you practice? (select the most practiced) | | | | | | (1) Cropping  (2) Mixed farming | | | | | | | | | (3) Beekeeping  (4) Livestock | | | | |
|  | Numbers of livestock | | Cattle | | | Sheep | | | | | | | Goats | | | Chicken | | | | Others |
|  |  |  |  | | |  | | | | | | |  | | |  | | | |  |
|  | Did you experience any type of natural hazard such as drought, extreme temperature, flooding etc. in the last five years? | | | | | | | | | | | | | | | | (1) No (2) Yes | | | |
|  | If Yes, for Q. 34, how often? | (1) Rarely (<Once in 5years)  (2) Sometimes (twice in 5years) | | | | | | | | | | (3) > twice in 5years  (4) Frequently (every year) | | | | | | | | |
|  | How do you rate the occurrence, amount and duration of rainfall in your locality? | | | | | | | | (1) Below average (decreasing)  (2) Uniform  (3) Above average (increasing)  (4) Undetermined | | | | | | | | | | | |
|  | How do you rate the intensity of temperature in your locality? | | | | | | | | (1) Below average (decreasing)  (2) Uniform  (3) Above average (increasing)  (4) Undetermined | | | | | | | | | | | |
|  | How is the wind direction you have been experiencing? | | | | | (10) significant change  (2) moderately change | | | | | | | | | | (3) No change  (4) Undetermined | | | | |

**II. Local adaptation and coping strategies: Household’s adoption status, perceived constraints to adaptation strategies**

1. The followings are the adaptation and coping strategies commonly practiced in Ethiopia. Thus, rank the mechanisms you have been practicing as ‘1,2,3,4’ if you use the mechanism ‘usually, sometimes, rarely and not at all’ respectively.

| **S.N** | **Adaptation Mechanisms** | **Rank** | **Coping strategies** | **Rank** |
| --- | --- | --- | --- | --- |
|  | Changing planting date |  | Change in consumption |  |
|  | Crop diversification |  | Receiving support through PSNP |  |
|  | Improved seed |  | Accessing credit |  |
|  | Non-farm activities |  | Selling livestock |  |
|  | Reducing stocking density |  | Renting out land |  |
|  | Improving livestock farm |  | Seasonal migration |  |
|  | Employed small scale irrigation |  | Remittance |  |
|  | Tree planting |  | Others, if any ranked as the 1^st^ three |  |
|  | Soil and water conservation |  |  |  |
|  | Soil fertility management |  |  |  |
|  | Drought-tolerant crops |  |  |  |
|  | Others, if any could be ranked as the first three |  |  |  |

1. Describe why you prefer to adopt the first three ranked strategies (depending on the question number 23) -------------------------------------------------------------------------------------------------------------------------------------------------------------------------------------------------------------------------------------------------------------------------------------------------------------------------------------------------------------------------------------------------------------------------------------------------
2. Identify the most important constraints to your adaptation strategies---------------------------------------------------------------------------------------------------------------------------------------------------------------------------------------------------------------------------------------------------------------
3. Describe the relationship between local livelihoods stressor/s (you believed as external to local causation) and adaptation constraints
4. Which one the following global environmental change processes have been affecting your adaptation options? climate change or structural land use dynamics? Why and how, explain the reasons and the processes? -------------------------------------------------------------------------------------------------------------------------------------------------------------------------------------------------------------------------------------------------------------------------------------------------------------------------------------------------------------------------------------------------------------------------------------------------------------------------------------------------------------------------------------------
