# Supplementary material for: Local adaptation and coping strategies to global environmental changes: Portraying agroecology beyond production functions in southwestern Ethiopia
Source: PLoS One. 2021 Aug 12;16(8):e0255813. doi: 10.1371/journal.pone.0255813 (PMC8360511; doi:10.1371/journal.pone.0255813)
Supplement: S2 Text — (DOCX) [file pone.0255813.s002.docx]

**S2 Text. English language focused group discussion protocol developed to collect data from elderly small-scale farmers to appraise local adaptation and coping strategies to global environmental changes across agroecology in Southwestern Ethiopia (DOCX)**

1. Global environmental change processes and implications on local adaptation strategies
   - 1. **Issues of land use land cover dynamics**

- What is land and land resource for small-scale farmers in your area?
- Traces on land tenure system, drivers and impacts of land use dynamics
- Trends of small-scale farmers’ access to land resource, local internal socio-economic and demographic dynamics, and external drivers in land use dynamics.
- Are there large-scale investment activities in your area? Who are investors in your area? What are the criteria to be investor? How do you evaluate their role and effects on local community and physical environment?
- How do you describe your relationships with investors and investment activities?
- Explain the main impacts of large-scale agriculture investment on your livelihoods?
- It could be either negative or positive
- Impacts difference among communities having different livelihood system (impacts on farmers, gatherers, hunters)
- Displacement of people
  - 1. **Issues of climate change and variability**
- Trends and variability conditions of rainfall and temperature
- How do you describe the impact of climate change on your adaptation strategies?
  - 1. **Simultaneous processes and impacts**
- How do you express climate change/variability and land use land cover dynamics in your area?
- Causes of the changes
- Natural increase (Rapid population growth and associate demand of land for agriculture, land fragmentation, overutilization of natural resources
- Socio- economic and political factors
- How do you express their impacts on small-scale farmers’ livelihoods?
- Are the impacts simultaneous and equal?
- Which stressor is severe in context of your livelihoods and why?
- How do you express the processes? Are they external or internal to local community? Why?

1. The current status and changing aspects in adaptations strategies of the local community

- Describe the trends of adaptation strategies;
- compare the efficiency of past and present strategies
- Causal linkage with dynamics in political and socio-economic situations and biophysical environment/and climate change.
- Currently, what mechanisms are mostly adopted? and how do you describe the present adaptation strategies?
- What are the implications of changes in the strategies on your livelihood system?
