# Supplementary material for: Local adaptation and coping strategies to global environmental changes: Portraying agroecology beyond production functions in southwestern Ethiopia
Source: PLoS One. 2021 Aug 12;16(8):e0255813. doi: 10.1371/journal.pone.0255813 (PMC8360511; doi:10.1371/journal.pone.0255813)
Supplement: Attachment — Submitted filename: PONE-D-21-14280 Overall comments.doc [file pone.0255813.s003.doc]

Title: Appraising local adaptation and coping strategies to global environmental changes: Portraying agroecology beyond production functions in Southwestern Ethiopia

**PONE-D-21-14280**

Overall comments: The manuscript is technically very sound and fit for publication in Plos one journal. The authors described all the sections very well, but the discussion needs to be substantially improved. The authors presented all the portions well clearly with novel information and according to the journal format. According to my opinion I strongly recommend this manuscript for publication in Plos One. I am fully satisfied on authors work for providing new insights in this field to the research communities. It is equally beneficial for farmers and scientific community to startup a new research program by the adaptation and application of new strategies that more suitable and more beneficial under climatic changing environment.

Thoroughly check the manuscripts and remove all the spelling mistakes. On the other hand, cross check all the references that quoted in the text with reference list.

Line No : 709 correct the spelling (Acknowledgements)

Line No: 716 Replace Reference with References
