# Supplementary material for: Local adaptation and coping strategies to global environmental changes: Portraying agroecology beyond production functions in southwestern Ethiopia
Source: PLoS One. 2021 Aug 12;16(8):e0255813. doi: 10.1371/journal.pone.0255813 (PMC8360511; doi:10.1371/journal.pone.0255813)
Supplement: Attachment — Submitted filename: Response to Reviewers.docx [file pone.0255813.s004.docx]

**Response to Reviewers**

**[Cover Letter]**

Dear Editor-in-chief,

We appreciate you and the reviewers for your precious time in reviewing our paper and providing valuable comments. It was your valuable and insightful remarks that led to possible improvements in the current version. We have carefully considered the comments, incorporated the suggestions, and tried our best to address every one of them. We hope the manuscript after careful revisions meet your high standards. Specifically, we have carefully considered removing the grammatical problems in this manuscript, and an exhaustive correction was made in this revised version. However, these grammatical corrections were too much that we didn’t include in this response letter, and in the ‘Manuscript Revised with highlighted change’.

This response letter consists of both general and detailed comments provided by all the reviewers. We provide the point-by-point responses for the detailed comments and suggestions. We look forward to hearing from you in due time regarding our resubmission, and to respond to any further questions and comments you may have.

Sincerely,

Gemechu Y. Ofgeha (corresponding author)

[dandigemechu11@gmail.com](mailto:dandigemechu11@gmail.com)

PhD scholar, Department of Geography and Environmental Studies

Addis Ababa University, Ethiopia

**Responses**

**Editor Comments:** Dear authors, I would suggest that you follow the reviewers' comments and suggestions. The paper has many weaknesses including results, discussions, formatting and English related.

**(Response):** We appreciate you and the reviewers for your precious time in reviewing our paper and providing valuable comments. We have carefully considered the comments, incorporated the suggestions, and tried our best to address every one of them. We hope the manuscript after careful revisions meet your high standards. Specifically, we have carefully considered removing the grammatical problems in this manuscript, and an exhaustive correction was made in this revised version. However, these grammatical corrections were too much that we didn’t include in this response letter, and in the ‘Manuscript Revised with highlighted change’

**Reviewer #1**

**(Comment:** **General)** The manuscript is well written and justified with reference to previous studies. The work is novel and nicely compiled. However, few minor corrections are required before further processing.

**(Response):** Thank you very much. We have incorporated the suggested correction in all parts of this paper.

**(Comment:** **P2: L26-58) “Abstract is way too long, there is a need to concise it according to the journal requirements.”**

**(Response):** Thank you very much. We have revised the abstract based on your comments.

**(Comment:** **General) “Formatting of all the sections should be rechecked.”**

**(Response):** Thank you. We have rechecked all sections of the paper based on the journal’s format.

**(Comment:** **General) “Headings should be according to the journal's format.”**

**(Response):** Thank you for the suggestions. We have made revisions for the heading and subheadings as follows:

- Conceptual frameworks (**page 5: Line 137**)
- Research methods and materials (**page 8: Line 238**)

**(****Comment:** **P27: L840-1009) “References need to be uniform in formatting.”**

**(Response):** Thank you very much. We have checked the references for its consistency.

**(Comment: P24: L742) “Conclusion of the study should not be more than a paragraph or two. It should only focus on main outcome of the study”**

**(Response):** Thank you for the suggestions. We have tried to condense the conclusions but it is difficult to conclude for all specific objectives within one or two paragraphs.

**Reviewer # 2**

**(Comment: General) “Thoroughly check the manuscripts and remove all the spelling mistakes.”**

**(Response): Thank you for the suggestions. We have made revisions accordingly.**

**(Comment:** **P27: L840-1009) “cross check all the references that quoted in the text with reference list.”**

**(Response):** Thank you very much. We have rechecked all the citations in the texts against the reference list. We found that the citation on the **Page 13: Line 399 and page 17: Line 490** was not referenced. Now it is included in the references list as follows:

39. Mbang A, Owino J, Kones J, Meffeja F, Dibog L. Evaluation of Ensiled Brewer’ s Grain in the Diet of Piglets by One Way Multiple Analysis of Variance, MANOVA. 2007.

41. Pallant J. Survival Manual:A step by step guide to data analysis using SPSS. 4th ed. New York, USA: Open University Press; 2010.

**(Comment: P21: L614) “Discussion section needs improvements.”**

**(Response):** Thank you for the comments. We have made revisions that hopefully this part after careful revisions meet the required standard.

**(Comment: P27:L833) “Line No: 709 correct the spelling (Acknowledgements)”**

**(Response):** Thank you very for the suggestion. We have revised.

**(Comment: P27: L840) “Line No: 716 Replace Reference with References”**

**(Response):** Thank you for the suggestion. We have revised.

**Reviewer #3**

**(Comment: General) “The article needs to improve according to format”**

**Response:** Thank you very much for the suggestion. We have revised the manuscript according to the journal’s format. Several formatting errors such as font size, text formatting, and the heading and subheadings were corrected in this revised version.

**(Comment:** **General) “author should clear the results and discussion regarding the statistical analysis.”**

**(Response):** Thank you very much. We have carefully considered the statistical assumptions and rules in the analysis applied in this study. It is our pleasure to accept you, in case if you have any specific comments/ suggestions regarding the statistical analysis.

**(Comment:** **P11: L337-339) “There should evidence from where collect the data.** **Should mention related references”**

**(Response):** Thank you very much for the comments. We have accepted all the comments, and rewritten this part to be clear with the sources of data as follows: The small-scale farmers’ household heads, and the focus group discussants drawn from local notoriety individuals and the elderly in the Anger watershed, and experts from relevant zonal and districts offices in the east Wallagga administrative zone were the primary sources of data (**P11: L337-339)**

**(Comment:** **General) “Grammatically mistakes should be removed”**

**(Response):** Thank you very much. We have carefully considered the comments regarding the grammatic problems in this manuscript, and an exhaustive correction was made in this revised version. However, these Grammarly corrections were too much to include in this response letter.
