# Supplementary material for: Local adaptation and coping strategies to global environmental changes: Portraying agroecology beyond production functions in southwestern Ethiopia
Source: PLoS One. 2021 Aug 12;16(8):e0255813. doi: 10.1371/journal.pone.0255813 (PMC8360511; doi:10.1371/journal.pone.0255813)
Supplement: Attachment — Submitted filename: Final PONE-D-21-14280 R1 comments.doc [file pone.0255813.s005.doc]

Title: Local adaptation and coping strategies to global environmental changes: portraying agroecology beyond production functions in southwestern Ethiopia

PONE-D-21-14280R1

Overall comments: The manuscript is technically very sound and study presents the results of original research that most fit for publication in Plos one journal. All the analyses/methods described in this study having a high technical standard and are described in sufficient detail. Over all article presented in a good way and meets the journal requirements. On the other hand, The authors presented all the sections in an appropriate fashion and meets all applicable standards for further processing of the article.

According to my opinion I strongly recommend this manuscript for publication in Plos One. I am fully satisfied on authors work for providing new insights in this field to the research communities. It is equally beneficial for farmers and scientific community to startup a new research program by the adaptation and application of new strategies that more suitable and more beneficial under climatic changing environment.

Comments for authors

i) Conclusion section is too lengthy please try to shorten it if possible
ii) Carefully cross check all the references that quoted in the text with reference list
iii) Over all article is suitable/fit for publication in Plos One Journal
