# Supplementary material for: Local adaptation and coping strategies to global environmental changes: Portraying agroecology beyond production functions in southwestern Ethiopia
Source: PLoS One. 2021 Aug 12;16(8):e0255813. doi: 10.1371/journal.pone.0255813 (PMC8360511; doi:10.1371/journal.pone.0255813)
Supplement: Attachment — Submitted filename: Response to Reviewers.docx [file pone.0255813.s006.docx]

**Response to Reviewers**

**[Cover Letter]**

Dear Editor-in-chief,

Once again, we appreciate you and the reviewer for your precious time in reviewing our paper and providing valuable comments. It was your valuable and insightful remarks that led to possible improvements in the current version. We have carefully considered the comments, incorporated the suggestions, and tried our best to address every one of them. We hope the manuscript after careful revisions meet your high standards.

This letter consists the response on the comments regarding the journal requirements and the editor’s and reviewers’ comments on the manuscript. We look forward to hearing from you in due time regarding our resubmission, and to respond to any further questions and comments you may have.

Sincerely,

Gemechu Y. Ofgeha (corresponding author)

[dandigemechu11@gmail.com](mailto:dandigemechu11@gmail.com)

PhD scholar, Department of Geography and Environmental Studies

Addis Ababa University, Ethiopia

**Responses**

**(Comments on the Journal Requirements):** Please review your reference list to ensure that it is complete and correct. If you have cited papers that have been retracted, please include the rationale for doing so in the manuscript text, or remove these references and replace them with relevant current references. Any changes to the reference list should be mentioned in the rebuttal letter that accompanies your revised manuscript. If you need to cite a retracted article, indicate the article’s retracted status in the References list and also include a citation and full reference for the retraction notice.

**(Response):** Thank you for the suggestion. We have carefully considered regarding the references of this manuscript. Now, we have ensured that it is complete and correct. Moreover, the issue of retracted articles is fully considered in this revision. Thus, we approved that no retracted sources were referenced in this manuscript.

**(Editor Comments):** Dear authors, please address the final remarks of the reviewers.

**(Response):** We appreciate you and the reviewers for your precious time in reviewing our paper and providing valuable comments. We have carefully considered the comments, incorporated the suggestions, and tried our best to address every one of them. We hope the manuscript after careful revisions meet your high standards.

**Reviewer #1**

**No comment provided**

**Reviewer # 2**

**(Comment:** **General) “Conclusion section is too lengthy please try to shorten it if possible.”**

**(Response):** Thank you for the suggestions. We have tried to condense the conclusions again. However, our concern is that trying to shorten more will result in missing to conclude for all specific objectives of the study.

**(Comment:** **General) “Carefully cross check all the references that quoted in the text with reference list.”**

**(Response):** Thank you very much for the reminder. We have carefully rechecked all the citations in the texts against the reference list.

**(Comment: General) “Over all article is suitable/fit for publication in Plos One Journal.”**

**(Response):** Thank you very much for the recommendations. Yours and other reviewers’ valuable comments have pivotal role for the fitness of this manuscript.
